# The ineligibility barrier for international researchers in US academia

**DOI:** 10.1038/s44319-023-00053-x

**Published:** 2024-01-23

**Authors:** Mikael H Elias, Kitty Sompiyachoke, Facundo M Fernández, Shina Caroline Lynn Kamerlin

**Affiliations:** 1 University of Minnesota, Minneapolis, USA; 2 Georgia Institute of Technology, Atlanta, USA

**Keywords:** Careers

## Abstract

Many grants from US funding agencies are restricted to US citizens or permanent residents which negatively impacts the career options for foreign scientists working in the USA.

**Figure:**
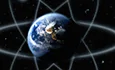

The USA presents many opportunities for academics with one of the highest education expenditures, resulting in excellent higher education, numerous highly-ranked universities, and first-rate research, all of which creates a prime environment for collaboration and academic growth. As such, the USA has long successfully attracted a significant number of foreign-born students: in 2006, the country drew 22% of all international students globally (Kim, 2012). In 2022–23, more than one million international students were enrolled at US colleges and universities, 55% of whom were majoring in STEM. US academia is also attractive to foreign-born researchers and faculty: a 2020 National Science Board report highlights that 49% of US-trained postdoctoral associates and 29% of full-time science and engineering faculty are foreign-born. Again, most of the foreign-born faculty are in STEM fields (Kim and Jiang, 2021).

**Figure:**
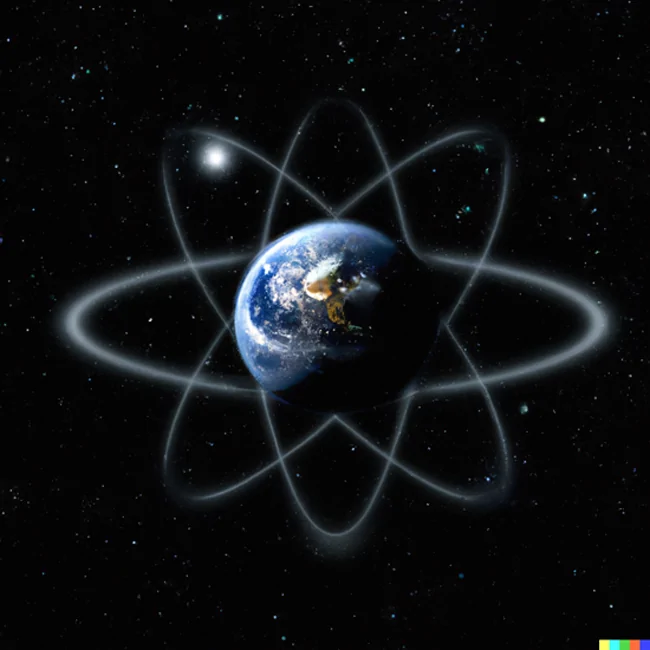
Science is Global and funding should respect this. Image generated by DALL-E.

The labor of such scientists strengthens research and innovation across the board: the USA dominates, for instance, representation among Nobel Laureates, and ~40% of these, are, in turn, foreign-born based on an analysis by the National Foundation for American Policy. Immigrants are also responsible for an aggregate 36% of US innovation, according to the National Bureau of Economic Research. Comparing the USA to other scientific/engineering powerhouses, the UK similarly benefits from immigrant scientists: according to the Royal Society, more than 60% of UK postdoctoral researchers have come from overseas. However, there is significant concern about new immigration requirements, which sets the minimum salary requirement for a work visa above that of a typical postdoc salary. In comparison, net migration is out of rather than into Germany, and similar concerns have been raised in Japan about the shortage of foreign scientists. It is worth noting though the language advantage that English-speaking countries such as the USA and UK have when it comes to recruiting foreign talent. However, there are also proactive attempts to creative attractive conditions, for instance, in Germany, through the DAAD program or support from the Humboldt Foundation to recruit overseas talent.

Given their obvious academic and innovation contributions to US academia and economy, one would expect that foreign-born researchers would be welcomed with open arms. In practice, however, they continue to face significant barriers to enter and participate in the US research enterprise. The most commonly discussed obstacles are the various forms of visa barriers faced by immigrant researchers, which have also been leveraged as a form of control of researchers in already vulnerable and precarious positions (Fleming, 2022). Moreover, problems with obtaining working visas can limit foreigners’ career options outside academia, should they wish to continue their work in the USA. These visa hurdles further hurt the economy, as they push US-based employers to relocate foreign talent. The situation remains challenging under the Biden administration, which recently proposed changes that continue to restrict H-1B work visas using similar restrictive language as under the Trump administration (the H-1B visa is a specific nonimmigrant visa for workers in specialty occupations such as science and engineering, as well as highly distinguished fashion models). The fact that the number of H-1B visas awarded annually is capped at currently 85,000 per fiscal year, compared to 780,884 entries for FY2024 creates further visa shortages and exacerbates what is effectively a skilled worker visa crisis.

While visa issues clearly create significant problems for researcher mobility, what is less discussed, but nevertheless a significant problem, is what we informally describe here as the ‘curse of ineligibility’. Even for foreign-born researchers—graduate students, postdocs and faculty—who do manage to secure appropriate visas and wish to pursue academic careers in the USA, there are significant inequalities in access to career-advancing funding opportunities.

Specifically, a considerable, and lesser known, hurdle is that non-US scholars are ineligible for a large fraction of research funding mechanisms that are available to US citizens and permanent residents, including Federal Student Aid. Many fellowships from both private foundations and federal agencies, as well as training grants, for instance from the NIH, are also not open for non-US scholars. This issue and its impacts have been previously highlighted in the case of medical students (Villamar and Albuja, 2022; David and Issaka, 2021; Radabaugh et al, 2019), but it also affects scholars in many other fields including STEM.

We are concerned that this absence of opportunity to compete for support is discouraging for many non-US young scholars and impacts US academic growth. We further surmise that the lack of financial support and training opportunities, created through such ‘eligibility barriers’ is significantly affecting the educational and professional development of these scholars, not least considering the crucial impact of securing early support for subsequent career progression in the hypercompetitive environment of academia. There has been significant discussion, for instance on social media, about the problems associated with such fellowships as markers of excellence for faculty hires, but this discussion has largely overlooked all the foreign-born students and postdocs who are not even eligible. We have lost count of the number of brilliant students we have trained who have not been able to apply for fellowships they are more than competitive for, simply because of their citizenship.

Problematically, such ineligibility issues do not end at graduation, or even after landing an academic position. They persist and are perhaps even amplified: non-US faculty are ineligible for many federal career development awards, except for NIH K99/R00 grants (David and Issaka, 2021). Although many such awards do also extend eligibility to permanent residents, becoming a permanent resident is a costly process that can take years and only a small fraction of all immigrants become permanent residents. Programs that are closed to foreign-born scientists include the young investigator programs from the Army Research Office, the Air Force or the Office of Naval Research. This is regrettable because these programs can springboard a scholar’s career and play a disproportionate weight in promotion and tenure discussions, which often do not consider the fact that foreign-born scientists are ineligible. This, in turn, disenfranchises promising foreigners who would otherwise have so much to contribute to US research and innovation, by not allowing them to compete on their own merits. It also creates a double standard as these student researchers are recruited to train and work in the USA only to have crucial career doors closed on them.

As many of these programs are federally sponsored, changing the eligibility criteria will be challenging and time-consuming. Yet, it is important to recognize that despite these additional difficulties and fewer opportunities, non-US scholars are held to the same evaluation criteria as US citizens and permanent residents at every stage of their careers. We believe that it is important to rigorously consider this smaller opportunity pool during evaluations for support and for positions. The foreign-born status could also be considered as one of the parameters for planning faculty retention programs (Oka et al, 2022).

We note that the foreign-born status creates barriers to alumni beyond research, and can prohibit their ability to teach in some States, or to apply for licensure (Mahon, 2010). Importantly, visa status represents an additional, significant barrier to finding work outside of academia (Goodwin et al, 2023). Overall, we echo a recent call for the NIH to revisit the eligibility criteria based on US citizenship (David and Issaka, 2021) and call for a global reflection for all funding agencies, federal or private. Given the importance of foreign-born students, faculty and professionals to US academia, such changes could further amplify their contribution to the US economy, something from which we will all benefit.
